# Screening for adolescent alcohol and drug use in pediatric health-care settings: predictors and implications for practice and policy

**DOI:** 10.1186/1940-0640-7-13

**Published:** 2012-08-16

**Authors:** Stacy Sterling, Andrea H Kline-Simon, Charles Wibbelsman, Anna Wong, Constance Weisner

**Affiliations:** 1 Division of Research, Kaiser Permanente Northern California, 2000 Broadway, 3rd Floor, Oakland, CA, 94612, USA; 2 Kaiser Permanente Northern California Medical Group, San Francisco Medical Center, 2200 O’Farrell Street, San Francisco, CA, 94115, USA; 3 Kaiser Permanente Northern California Medical Group, Oakland Medical Center, 3505 Broadway, Oakland, CA, 94611, USA; 4 Department of Psychiatry, University of California, San Francisco, 401 Parnassus Avenue, San Francisco, CA, 94143, USA

**Keywords:** Adolescent, Screening, Brief intervention, Alcohol, Drugs, Primary care, Behavioral, SBIRT

## Abstract

**Objective:**

This paper used data from a study of pediatric primary care provider (PCP) screening practices to examine barriers to and facilitators of adolescent alcohol and other drug (AOD) screening in pediatric primary care.

**Methods:**

A web-based survey (N = 437) was used to examine the influence of PCP factors (attitudes and knowledge, training, self-efficacy, comfort with alcohol and drug issues); patient characteristics (age, gender, ethnicity, comorbidities and risk factors); and organizational factors (screening barriers, staffing resources, confidentiality issues) on AOD screening practices. Self-reported and electronic medical record (EMR)-recorded screening rates were also assessed.

**Results:**

More PCPs felt unprepared to diagnose alcohol abuse (42%) and other drug abuse (56%) than depression (29%) (p < 0.001). Overall, PCPs were more likely to screen boys than girls, and male PCPs were even more likely than female PCPs to screen boys (23% versus 6%, p < 0.0001). Having more time and having other staff screen and review results were identified as potential screening facilitators. Self-reported screening rates were significantly higher than actual (EMR-recorded) rates for all substances. Feeling prepared to diagnose AOD problems predicted higher self-reported screening rates (OR = 1.02, p <0.001), and identifying time constraints as a barrier to screening predicted lower self-reported screening rates (OR = 0.91, p < 0.001). Higher average panel age was a significant predictor of increased EMR-recorded screening rates (OR = 1.11, p < 0.001).

**Conclusions:**

Organizational factors, lack of training, and discomfort with AOD screening may impact adolescent substance-abuse screening and intervention, but organizational approaches (e.g., EMR tools and workflow) may matter more than PCP or patient factors in determining screening.

## Introduction

Alcohol and other drug (AOD) problems are major causes of mortality and morbidity, and these problems often begin in adolescence [1]. Pediatric primary care providers (PCPs) are ideally placed to identify AOD problems in their adolescent patients and intervene before the problems become more serious. Unfortunately, behavioral health fields have failed to persuade PCPs and health systems to implement screening, brief intervention, and referral to treatment (SBIRT) for adolescents despite a mature body of evidence demonstrating the effectiveness of SBIRT for alcohol problems in adult primary care, and less robust but growing evidence suggesting that brief intervention models of AOD services are similarly effective for adolescents.

In this paper, we examine barriers to and facilitators of AOD screening and intervention in pediatric primary care using data from a study that examined pediatric PCP practices and attitudes toward screening and treatment for adolescent AOD use in a large integrated health-care delivery system. The study’s intent was to inform the development of strategies to facilitate adolescent AOD screening.

### AOD use and adolescent health

Alcohol and other drug problems are a major cause of morbidity and mortality among youth [2-4] and are a significant public health problem. More common than AOD disorders, however, and quite prevalent among adolescent primary care patients, is less severe but still risky AOD use. A survey of adolescent patients in a pediatrics setting found prevalence rates for AOD abuse to be 14.8% [5]. Adolescent AOD misuse co-occurs frequently with mental health problems and other conditions and behaviors that confer risk for unfavorable health, delinquency, HIV [6], poor academic performance, and suicide [7]. Considered alongside emerging evidence of the heightened vulnerability of the developing adolescent brain to the harmful effects of AOD use [8,9], it seems clear that early identification and treatment may prevent adverse long-term medical and mental health outcomes.

### Missed opportunities for problem identification in pediatric primary care

Medical visits provide critical opportunities to detect AOD problems [10], and PCPs may be especially effective agents to do so [11]. While many PCPs may worry that patients do not wish to discuss AOD use during check-ups, a recent national survey of teens and parents actually found high receptivity to screening and intervention by PCPs [12]. Brown and Wissow [13] found that adolescents had more positive perceptions of care when their PCP discussed “sensitive” topics with them, including AOD use.

Although all adolescents with AOD problems may not have access to or seek regular medical care, studies suggest that these adolescents may be just as likely as those without problems to visit a PCP [14]. Among adolescents seeking AOD treatment in the health system in which this study took place, 81% had a primary care visit in the year before treatment, and 90% had one in the two years prior to intake (unpublished observation, presented as “*The role of primary care in addressing adolescent substance use: screening, treatment, and coordination” in Los Angeles on* 6/22/07 at the California Society of Addiction Medicine symposium: "Not Just Small Adults: New Insights on Adolescent Brain Development and Implications for Adolescent Substance Abuse Treatment Conference"). In addition, AOD use is often associated with other medical and mental health problems. Adolescents with AOD problems have been found to have more medical and psychiatric problems than those without AOD problems [15,16], and, although competing clinical priorities may reduce the likelihood of addressing AOD disorders, comorbidities may also give providers an entree for discussing AOD use with patients.

### Adolescent AOD screening recommendations

Adolescent health experts have long called for more and better AOD screening in pediatrics [17-19]. Several national organizations, including the US Preventive Services Task Force, have developed clinical guidelines for adolescent health care that specifically recommend screening for AOD use. The American Academy of Pediatrics’ *Bright Futures* and the American Medical Association’s *Guidelines for Adolescent Preventive Services* both recommend that youth aged 11 years and older should be screened for AOD use at each annual preventive health visit [20-23].

### Suboptimal screening practices

Despite these recommendations relatively few PCPs screen adolescents according to guidelines [24-26]. An American Academy of Pediatrics’ survey found that only 45% of fellows routinely screened young patients for alcohol use, and only 16% reported using standardized instruments [24]. There are many possible reasons for these low levels of screening, including organizational and systemic factors such as insufficient time and staff resources [10], inadequate training in residency programs [27], concerns about intrusiveness or patient embarrassment [28,29], and low levels of self-efficacy and confidence about discussing AOD use [30-32].

Research also suggests that, based on clinical impressions alone, PCPs may fail to detect AOD problems among adolescent patients. One study found that only 2% of the 16% of adolescents with diagnosed AOD disorders were correctly identified by physicians as having a substance-use problem [33]. Even when AOD screening occurs, identification, intervention, and/or referral to specialty care are not guaranteed. A study by Stevens et al. [34] that screened adolescents for AOD disorders in the waiting room found that doctors who received the results prior to seeing the patient improved identification significantly compared with those who received results after the visit; however, they still failed to recognize problems in 27% of the patients whose screening results indicated an AOD problem. In another study, Hassan and colleagues [35] examined pediatricians’ perceptions of the severity of patients’ AOD problems and their practices with regard to follow-up interventions. They found that, while 14% of the sample scored ≥2 on the CRAFFT (**C**ar, **R**elax, **A**lone, **F**orget, **F**riends, **T**rouble) screening tool, indicating a likely AOD problem, providers’ diagnostic impressions led them to identify only 4.8% of the patients (N = 2034) with problem use. Moreover, almost 20% of those perceived by the providers to have an AOD problem still did not receive a recommendation for an active intervention [35]. Another study of adolescents entering specialty AOD treatment in a large health-care system found that fewer than 20% of new patients were identified and referred to treatment by their medical provider [16]. These findings suggest that youth with AOD problems are not always well-served by the health-care system or providers, and that more work is needed to improve detection and treatment.

### SBIRT and adolescents

As a nationally recognized public health approach to AOD problems, SBIRT is strongly supported by the National Institute of Alcohol Abuse and Alcoholism (NIAAA), the National Institute on Drug Abuse (NIDA), and the Substance Abuse and Mental Health Services Administration (SAMHSA), as well as by national medical organizations including the American Academy of Pediatrics [17]. A solid body of research developed over the past two decades suggests that SBIRT is efficacious for reducing risky AOD use in adolescents and that the principles and techniques underlying SBIRT (e.g., motivational interviewing) may in fact be particularly well-suited to the developmental stage of adolescence [36,37]. Several studies have demonstrated the efficacy [38-40], effectiveness [41,42], and feasibility [43] of screening and brief intervention for adolescents on a wide range of outcomes, including reducing binge drinking, cannabis use, drinking and driving, smoking, emergency department use, and other harmful behaviors [36,38,40,44-53].

### SBIRT in pediatric primary care

Research examining SBIRT for adolescents in pediatric primary care settings has also shown promising results. Ozer et al. [54] found that a brief alcohol intervention by pediatric PCPs to 14- and 15-year-olds reduced risky drinking [54]. De Micheli and colleagues [55] found that a brief intervention delivered by pediatricians in an adolescent primary care clinic resulted in a reduction in use of several substances, including marijuana, alcohol, inhalants, Ecstasy, and tobacco, in the intervention group compared with controls who received no intervention. Knight et al. [40] found promising results at three months in a pilot study conducted partially in primary care, with an intervention delivered by both pediatricians and nonphysicians; however the sample was small, attrition was significant, and they did not examine differences in effectiveness between the PCPs and non-PCPs. D’Amico et al. [56] examined the impact of a brief motivational intervention on AOD drug use for high-risk teens in a primary care clinic and found decreased use and increased self-efficacy at three months; however, this pilot study also suffered from a small sample size and low retention rates.

### Factors influencing screening

**Figure 1:**
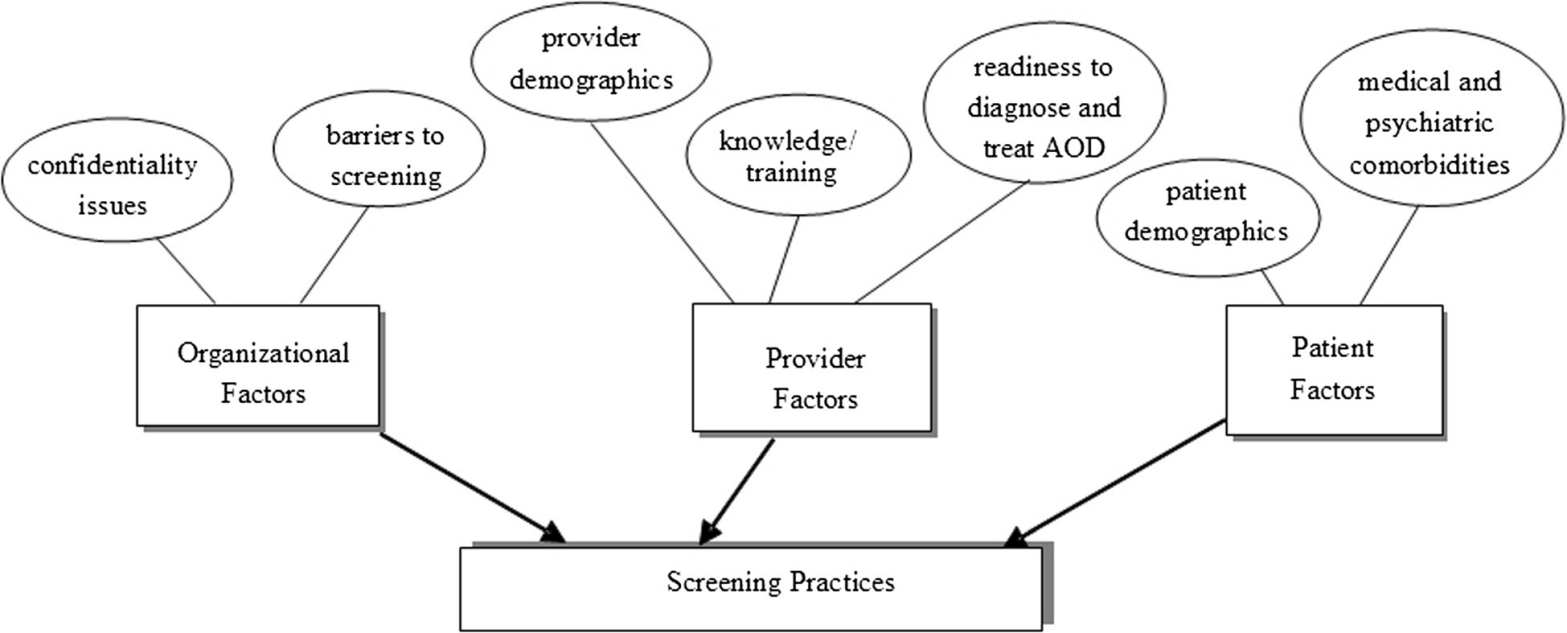
Provider, Patient, and Organizational Factors that Influence Screening and Brief Intervention Practices.

The literature is well-established on provider skills and attitudes, patient factors, and organizational factors that influence screening and brief intervention practices. These factors can be organized within a conceptual framework of provider, patient, and organizational factors (Figure 1).

Primary care physicians may fail to screen for alcohol use because of time constraints, lack of resources, lack of training, unfamiliarity with screening instruments, and attitudes toward particular health issues, including estimation of risk [57-59]. In a recent survey, members of the American Academy of Pediatrics reported the following barriers to providing services for child/adolescent behavioral problems: lack of time (77%), lack of training in treatment of such problems (65%), lack of confidence in their ability to address problems (62%), and lack of qualified specialty treatment providers to whom to refer patients (61%) [60]. A qualitative examination of barriers specific to adolescent AOD screening ranked the barriers as: 1) insufficient time, 2) lack of training to manage positive screens, 3) need to triage competing medical problems, 4) lack of treatment resources, 5) and “tenacious” parents who compromise confidentiality, and 6) unfamiliarity with screening tools [61]. Many of these barriers speak to increasing pressure on PCPs to perform more services within shorter appointment times.

That insufficient time is oft cited as a barrier to screening reflects the pressures faced by today's PCPs. Preventive health activities increase the average time spent with patients [10]: one study estimated that for a PCP to provide all the patients in a typical panel with all the services recommended by the US Preventive Services Task Force would require 7.4 hours per day [59]. Addressing behavioral health concerns has been found to significantly increase visit duration [62].

Providers also may not feel that behavioral health problems, particularly AOD problems, fall within their purview. A recent survey of pediatricians found that, while the majority (88%) felt that they should be responsible for identifying substance abuse among their patients, very few (21%) felt that they should be responsible for the treatment and management of those problems, preferring instead to refer such patients to other providers for treatment (90%) [63]. This is in spite of the fact that many primary care providers also express skepticism about the effectiveness of specialty AOD treatment. One survey found that only 21% of PCPs believed that interventions would be effective at least half the time [28]. Other provider factors that may predict screening include outcome expectancy, concerns about intrusiveness, negative attitudes about patients with alcohol problems, and level of confidence about screening and intervention skills.

Characteristics such as gender, age, and experience may also play a role in how well providers identify AOD use and intervene [64-66]. Some studies have found provider gender to predict screening practices, with women PCPs being more likely than men to provide preventive services, including screening patients for alcohol problems and using standardized instruments [30,65]. In a national survey of adolescent patients, Klein and Wilson found that patients were more likely to report having discussed health risk behaviors if their doctor was female [66]. Other studies found that younger providers report more screening than older ones [30,65], although this may actually reflect differences in training rather than age.

Patient demographic characteristics and medical and psychiatric conditions may also affect the likelihood of being screened and treated for AOD use, although this has not been well-studied in adolescents. A US survey of PCP screening of adults found that most primarily screen pregnant women and patients suspected of having a problem [67]. Gender differences have been found in how other psychosocial problems are diagnosed [68,69], and some studies suggest that female patients are less likely to be screened than male [70-72]. Older adolescents are more likely to be screened than younger [73,74].

The barriers to and facilitators of AOD screening have not been as well-studied in pediatrics as in adult medicine, and they have not been studied in the context of a health plan with electronic medical records (EMRs), which may reduce some of the previously reported barriers. This paper describes findings from a study that explored the approaches of pediatric PCPs to adolescent AOD screening and intervention in primary care. We explored factors that might either prompt PCPs to screen their patients or cause them to refrain from screening. We examined the prevalence of self-reported and EMR-recorded adolescent AOD screening in a population base of PCPs. We assessed the PCP factors (e.g., demographics, training, knowledge, and attitudes), patient factors (e.g., age, gender, co-occurring conditions, and other risk factors), and organizational factors (e.g., time constraints, confidentiality policies) that influence providers’ screening practices. We also examined PCPs’ perceived barriers to screening practices, and what mechanisms and approaches would facilitate screening. Finally, we examined PCP practices when adolescent AOD problems are identified. Findings could shed light on solutions to the persistent obstacles faced by those seeking to implement screening and brief intervention for AOD problems of adolescents in medical settings.

## Methods

### Setting

The study was conducted in the Kaiser Permanente Northern California (KPNC) health-care system; a large integrated health care delivery system serving over 3.4 million members (about 34% of the commercially insured population of the Northern California region), with approximately 450,000 members between ages 11 and 21. Kaiser Permanente Northern California has 48 medical facilities and employs approximately 607 pediatricians. In general, the membership is working- and middle-class, although several counties have contracted with KPNC to serve their Medicaid patients. The KPNC system provides integrated AOD and psychiatry services.

### Sample and procedures

The sample consisted of all KPNC PCPs based in pediatric or family practice departments who had at least 50 adolescents currently on their panel (N = 540). A provider’s “panel” consists of all members for whom the provider is the PCP.

Eligible providers were contacted via e-mail about an online survey and were invited to participate. The e-mail contained a hyperlink to the survey. All KPNC providers have access to e-mail and the organization’s intranet on the computers located in their offices; most also have access at home and the ability to read e-mail at their leisure. The survey software allowed participants to start and stop as desired. Passive consent was assumed if PCPs chose to access the survey, and they were offered a $50 gift certificate to reimburse them for their time. E-mail reminders were sent to nonresponders. After two reminders, research staff contacted the participant to administer the interview via telephone or to set up an in-person interview. Survey data were captured on a secure server at the Kaiser Permanente Division of Research and stored on password-protected computers. The study received approval from the Institutional Review Boards at KPNC and at the University of California, San Francisco.

### Measures

#### Survey data

The survey included four main sections: provider, patient, and organizational factors impacting AOD screening and provider screening practices.

#### Provider factors

We measured PCP age, gender, and ethnicity; medical specialty (general pediatrics, adolescent medicine); and years of experience (less than 10 or ≥10). We asked participants about the extent of their recent AOD training (training within the past 5 years versus none) and whether they felt satisfied with continuing medical education opportunities to stay current on issues of AOD and mental health problems in adolescents.

We examined levels of comfort with, and perceived expertise in, adolescent behavioral health problems by asking participants how difficult they felt it was to discuss alcohol abuse, drug abuse, depression, and risky sexual practices with their patients. They were asked how prepared they felt to diagnose patients with alcohol, marijuana, other illicit drug, and prescription drug problems and/or depression. The four questions related to readiness to diagnose AOD problems were combined to create a “prepared to diagnose” AOD composite score. These questions were first dichotomized (prepared versus not prepared to diagnose) and then summed to create a score ranging from 0 to 4 indicating the providers’ level of comfort in diagnosing AOD disorders (4 = very prepared). We also asked providers to what extent they felt knowledgeable about AOD use trends among adolescents and their opinion about the effectiveness of AOD specialty treatment.

#### Patient factors

These questions related to patient characteristics that were most likely to prompt PCPs to screen and address AOD problems. We asked participants if they were more likely to screen boys versus girls or older versus younger adolescents and which ethnic groups have higher rates of AOD problems. Potential warning signs (e.g., school problems, depression, legal problems, family conflict, parental AOD abuse, unusual sleeping patterns, and weight loss) that would prompt them to screen for AOD use were also examined. Regarding the types of substances their patients were using, the PCPs were asked which substances were most frequently misused among patients with AOD problems and which substance they felt posed the greatest risk to their patients. We also asked them to rank, in order of importance, the health conditions or risk factors for which their patients should be screened.

#### Organizational factors

We measured the organizational factors that PCPs felt were either barriers to or facilitators of adolescent AOD screening and treatment, such as time constraints, confidentiality issues, referral and treatment resources, proximity to mental health and AOD treatment, and perceptions as to which staff are best equipped to deliver such services. We asked them the reasons why they might refrain from discussing AOD use with their patients and what it would take to consistently screen every adolescent for substance use. We also asked whether they felt that organizational policies and state laws about confidential adolescent health services presented barriers to discussing AOD use with their patients.

#### Provider self-reported screening practices

Providers were asked to estimate both how often they screened their adolescent well-visit patients for AOD use and the percentage of adolescent well-visit patients they screened for the following: alcohol, drugs, tobacco, friends' AOD use, and riding in a car when the driver had used AOD. We asked whether, in addition to the AOD measures in the electronic medical record (EMR), they used standardized, evidence-based AOD screening instruments to assess their patients’ AOD use. They were asked about their usual practice if they suspected a patient had an AOD problem; i.e., whether they were likely to counsel patients themselves or refer them to someone else, and if the latter, to whom or what department were they likely to refer.

#### Electronic medical records

The KPNC network maintains its own EMR system, which integrates clinical and diagnostic data with appointments, registration, and billing for each encounter. Diagnoses and procedures are coded according to both ICD-9-CM and CPT4 classification systems. The records also include information from other data sources such as patient questionnaires filled out by clinicians. The “Teen Well Check” template is an integral part of every adolescent’s electronic chart. It records the results of physical exams and is the official documentation template for every adolescent well-visit. The template includes the “Teen Well Check Questionnaire,” a comprehensive checklist comprised of questions on a variety of health behaviors, including alcohol use (past year), marijuana and other drug use (ever), tobacco use (past year), close friends using AOD, driving with a driver who has used AOD, depression, and suicidality. The questionnaire is part of the standard recommended workflow for all adolescent well-visits within the KPNC health system and is completed by the adolescent prior to seeing the PCP for his or her physical exam.

We examined EMR data for each provider who participated in the study. The mean panel age for each provider was calculated. EMR-recorded screening rates were also calculated. Screening rates for each provider were defined as the percentage of the total number of adolescent patients who presented for a teen well-visit who were then screened for behavioral health problems. Screening was defined as the PCP having completed the Teen Well Check Questionnaire set of AOD-related questions.

#### Analysis

Basic frequencies and Pearson chi-square tests were used to describe the provider, patient, and organizational factors associated with screening. Paired t-tests were used to analyze differences in self-reported and EMR-recorded screening rates. Of the 437 providers who responded to the survey, 397 had at least one teen with a well-visit in the six months prior to the survey. The EMR-recorded screening rates were based on these visits. We built multivariable logistic regression models to examine the independent effects upon screening rates of average panel age, preparedness to diagnosis AOD problems composite score (0–3), and three barriers to screening: time constraints, concerns about adolescent confidentiality, and being uncertain that treatment is effective. Both self-reported and EMR-recorded screening rates were modeled. All analyses were conducted using SAS/STAT® software version 9.2 (SAS Institute, Inc., Cary, NC).

## Results

The web-based survey had an 81% response rate (N = 437). The mean age of PCPs in the sample was 45; 53% were white, 34% were Asian, 9% were Hispanic, and 4% were African American; 60% were female. The average age of patients in the PCPs’ panels was 14.5. The mean time since training was 17 years.

### Provider factors

PCPs reported that several factors influenced screening practices, including limited AOD screening and treatment knowledge and low self-efficacy/high sensitivity about addressing AOD problems, particularly compared with other behavioral problems. Nineteen percent of the PCPs reported that it was “difficult” or “very difficult” to discuss alcohol abuse with their patients; 22% found it difficult to discuss drug use, and 15% found it difficult to discuss depression (Table 1). Only 1% felt “not at all comfortable” discussing risky sexual practices with their patients. Many PCPs felt unprepared to diagnose AOD problems: 42% felt unprepared to diagnose alcohol problems, 37% felt unprepared to diagnose marijuana problems, and 56% felt unprepared to diagnose other illicit drug or prescription drug problems. In contrast, only 29% felt unprepared to diagnose depression among their patients (Table 2). Thirty percent reported that they did not discuss AOD use with their patients because “patients don’t tell the truth” about AOD use; 6% did not discuss it because they did not want “to frighten or anger” patients, and 5% did not discuss it because they might be seen as “questioning patients’ integrity.” Fourteen percent said that feeling “uncomfortable talking about AOD abuse” would prevent them from discussing AOD use with their patients (Table 2).

**Table 1:** Factors Influencing Screening

	**Percent of Providers (N = 437)**	**p-value**
**Difficult to discuss with patients:**		
Drug abuse	22	
Alcohol abuse	19	
Depression	15	< 0.05
**Unprepared to diagnose:**		
Other drug	56	
Alcohol	42	
Marijuana	37	
Depression	29	< 0.001
**Five most important risk factors to screen for:**		
Nutrition	72	
STDs	72	
Exercise	66	
Depression	59	
Alcohol use	40	< 0.001

**Table 2:** Barriers and Facilitators to Screening (N = 437)

**Barriers:**	**% Endorsing**
Time constraints	80
Adolescent confidentiality policies and regulations	32
Patients do not tell the truth	30
Uncertain treatment is effective	24
Uncomfortable talking about AOD use	14
Did not have sufficient information about referral options	12
Documentation of AOD in medical record could adversely affect patient	9
Do not want to frighten or anger patient	6
Questions patient's integrity	5
**Facilitators:**	**% Endorsing**
Extra time	76
Having an MA screen or clinician to review results	56
Having the screening done prior to seeing patient	51

Although only 14% reported receiving no instruction on AOD problems at all in the past five years, 48% were satisfied they were staying current on AOD problems and treatment. Over half the sample (52%) felt that AOD treatment was either not very, or not at all, effective. Those PCPs with <10 years of experience said they were less likely to talk to their patients about AOD use because of uncertainty about AOD treatment effectiveness, compared with those with more experience (32% versus 20%). Fewer female PCPs said that treatment was effective (25% versus 33%), and more reported that confidentiality was a barrier to discussing AOD use with their patients (35% versus 27%).

### Patient factors

There was variation as to which patients PCPs were most likely to screen and what conditions and risk behaviors prompted screening. Thirteen percent of PCPs reported being more likely to screen boys than girls, and male PCPs were even more likely than females to report that they were more apt to screen boys than girls (23% versus 6%, p < 0.0001). They reported perceptions of differences in the rates of AOD problems by ethnicity; e.g., 39% said that white adolescents had higher rates of AOD problems, followed by African Americans (37%), Hispanics (35%), Native Americans (14%), Pacific Islanders (6%), and Asians (4%). Sixty-two percent of PCPs said they were more likely to screen older than younger adolescents. In order of importance, the conditions they reported would trigger AOD screening were depression (94%), school problems (93%), weight loss (78%), sleeping problems (75%), family conflict (71%), and anxiety (71%). Eighty-six percent felt that AOD use was related to psychiatric comorbidity. When PCPs were asked to rank what they considered to be the most pressing conditions and risk factors to screen their patients for, alcohol use was ranked the fifth most serious risk; the other four were: 1) nutrition, 2) STDs, 3) exercise, and 4) depression (Table 1).

When asked which substances were more frequently misused among patients with an AOD problem, respondents reported alcohol (89%), marijuana (88%), party drugs (20%), methamphetamines (14%), prescription opiates (8 %), cocaine (7%), and inhalants (1%). They ranked the substances they considered most risky for their patients to use as heroin (90%), methamphetamine (86%), cocaine (85%), prescription opiates (75%), inhalants (74%), party drugs (72%), alcohol (28%), and marijuana (25%).

### Organizational factors

Eighty percent of respondents reported that time constraints would cause them to avoid discussing AOD use with patients, making it the number one barrier. Other reported barriers to screening were feeling that they did not “have sufficient information about referral options” (12%), and the fear that documentation of AOD use in medical record could adversely affect patients (9%). Many of the PCPs (32%) perceived adolescent confidentiality policies and regulations as a barrier to discussing AOD use and problems. The major facilitators they suggested for consistent screening were extra time (76%), using other providers to screen (56%), and having screening done prior to seeing the patient (51%) (Table 2).

### Provider self-reported screening rates and EMR-recorded screening rates

Only 5% of the PCPs reported using standardized evidence-based AOD screening instruments to assess patients’ AOD use. If they suspected a patient had an AOD problem, 93% reported they would refer them to someone or somewhere else for treatment, 91% reported that they would counsel the patient on the dangers of AOD use, and 42% said they would provide educational materials. Seventy-four percent said they would refer patients with AOD problems to mental health treatment, while 61% said they would refer them to specialty AOD treatment. We examined PCPs’ EMR-recorded screening rates among adolescents who had a well-child visit with them in the prior 6 months and found their self-reported screening rates were significantly higher than their EMR-recorded rates for all substances: alcohol (92% reported versus 65% EMR, p < 0.001); drugs (88% versus 64%, p < 0.001); and tobacco (92% versus 64%, p < 0.001) (Table 3).

**Table 3:** Provider Self-Reported and EMR-Recorded Screening Rates

	**Self-Reported Rates (N = 437)**	EMR-Recorded Rates (n = 397)*	**p-value**
**Screening Questions Asked:**			
Alcohol	92%	65%	<0.001
Drugs	88%	65%	<0.001
Tobacco	92%	66%	<0.001
Friends' alcohol and drug use	76%	66%	<0.001
In a car when driver used alcohol and/or drugs	47%	66%	<0.001

*Of the 437 providers who responded to the survey, 397 had a Teen Well Check visit in the six months prior to the survey from which the EMR rates are based.

### Independent predictors of self-reported and EMR-recorded screening

Participants who felt prepared to diagnose AOD problems were more likely to have higher self-reported screening rates (OR = 1.02, 95% CI = 1.01-1.03), while those who identified time constraints as a screening barrier were less likely to have higher self-reported AOD screening rates (OR = 0.91, 95% CI = 0.88-0.94). Concerns about confidentiality and uncertainty that treatment is effective were not significantly associated with self-reported screening rates. Those PCPs with an older average panel age were more likely to have higher EMR-recorded AOD screening rates (OR = 1.11, 95% CI = 1.07-1.16). (Table 4).

**Table 4:** Self-Reported and EMR-Recorded Screening Logistic Regression Models

	**Self-Reported Rates (N = 437)**		EMR-Recorded Rates (n = 397)*	
	**OR**	**CI**	**OR**	**CI**
Average panel age	1.01	0.98-1.05	1.11**	1.07-1.16
Prepared to diagnose AOD composite score	1.02**	1.01-1.03	1.00	0.99-1.01
Time constraints as barrier (yes versus no)	0.91**	0.88-0.94	1.01	0.97-1.05
“Uncertain treatment is effective” as barrier (yes versus no)	1.00	0.98-1.03	0.99	0.96-1.01
Confidentiality as barrier (yes versus no)	1.03	1.00-1.06	0.98	0.94-1.01

*Of the 437 providers who responded to the survey, 397 had a Teen Well Check visit in the six months prior to the survey from which the EMR rates are based

**p < 0.001.

## Discussion

As other studies have found [31,32], PCPs’ knowledge about AOD screening and treatment techniques, their self-efficacy in regard to addressing AOD use, and their discomfort with discussing AOD use were each identified as factors influencing screening practices. Results suggest they feel less prepared to diagnose alcohol than depression, they rate alcohol as more difficult to discuss than depression, and they are more comfortable talking about sexual practices than alcohol. Nineteen percent reported alcohol as being “difficult to discuss,” whereas only 1% reported “feeling uncomfortable” discussing risky sexual practices. Sensitivity about discussing AOD use has been cited as a barrier to screening among adult patient populations [75] because providers fear alienating patients or are hesitant to criticize culturally sanctioned behavior (drinking alcohol and, increasingly in some states, using marijuana). However, our findings suggest PCPs find alcohol and drugs difficult to discuss with adolescent patients even when their use is neither sanctioned nor legal. Training to destigmatize these topics and to increase PCP confidence and self-efficacy about discussing drinking and drug use would help address these problems. As found in other research, however [76], fewer opportunities for education about adolescent AOD use and assessment and treatment are available than providers would like; less than half were satisfied that they were staying current on AOD-related health topics. Fortunately, several new US training grants and medical school loan repayment programs target AOD screening and treatment education for PCPs, emphasizing the integration of mental and physical health and the treatment of vulnerable groups such as those with AOD problems [77]. Moreover, many organizations, including the American Academy of Pediatrics and the Substance Abuse and Mental Health Services Administration (SAMHSA), have initiatives to increase screening for adolescents, improve the environment for addressing alcohol as part of health care (as with smoking), and facilitate provider familiarity with AOD use and problems [17].

Patient characteristics also influenced attitudes towards screening. The majority of PCPs said they were more likely to screen boys than girls and were more likely to screen older than younger patients. They also felt that teens of different ethnic groups had different rates of AOD problems, with white, African American, and Hispanic youth perceived as having higher rates of use than other ethnic groups. The PCPs surveyed in this study clearly recognized the role of psychiatric comorbidity; 86% saw it as related to AOD problems. Depression was reported to be the most salient trigger for AOD screening, followed by school problems, weight loss, sleeping problems, family conflict, and anxiety. The providers ranked alcohol as only the fifth most pressing risk factor warranting screening, after nutrition, STDs, exercise, and depression; drug use did not make the top five risk factors. All these conditions and behaviors are compelling risk factors in adolescent health; thus, PCPs must balance a multitude of competing priorities in a visit [59].

As other studies have found [10], time constraints emerged as an important barrier to screening. The presence of an EMR to help standardize procedures and instruments did not obviate that key issue. Other factors, such as uncertainty about AOD treatment effectiveness, concerns about the potentially detrimental effect of such discussions on the provider-patient relationship, and discomfort discussing AOD issues were identified as significant barriers by fewer PCPs. One-third of the PCPs cited confidentiality as a barrier to screening; they struggle with the challenges involved in addressing AOD problems within the context of a confidential adolescent visit, balancing the need to maintain a relationship of trust with their patients, and the occasional need to break confidentiality and reveal AOD use to parents, particularly when problems may be severe enough to warrant referral to specialty treatment.

We found few independent predictors of screening, either self-reported or EMR-recorded. Those PCPs with panels of higher average patient age had higher EMR-recorded screening rates, while those who reported being more prepared to diagnose AOD problems had higher odds of increased self-reported rates, and those who endorsed time constraints as a barrier to screening were more likely to have lower self-reported screening rates. The discrepancy between self-reported and EMR screening rates is troubling but not unexpected; other research has found over-reporting of preventive clinical activities related to similarly “sensitive” topics [78]. The less-than-optimal EMR-recorded rates suggest that, even with systematic screening, an AOD screening tool embedded in a health system’s EMR, and standardized workflow, more effort is needed to ensure that AOD screening is a component of all adolescent well visits (and all nonemergency visits, many would argue). Conversely, the fact that nearly two-thirds of all teens are being screened during well visits, a higher rate than that found in other studies [24], suggests the value of system-level strategies to facilitate screening. These findings, coupled with reports from PCPs that AOD knowledge and self-efficacy as well as organizational issues such as time limitations and staffing influenced their screening practices, suggest the benefits of exploring a model of systematic screening that employs both standardized evidence-based screening instruments *and* clinicians trained in AOD screening and assessment.

Based on the survey findings, we conducted a pilot study of adolescent screening, brief intervention, and referral to treatment (SBIRT) in a large general pediatric clinic within the KPNC health system. The intervention sought to address organizational barriers identified in the survey, such as PCPs’ well-founded concerns about the additional time required to address behavioral problems in time-limited well-visits [62] and about having other personnel conduct the screening and intervention (if needed). At the same time, it addressed, directly or indirectly, some of the more complex provider-level factors that could impede screening (for example, low levels of self-efficacy and high levels of discomfort about discussing AOD problems) and allowed for referral for broader behavioral health problems.

Compared with usual care, which does not provide formal “in-house” behavioral health services in general pediatrics departments, this pilot intervention provided as a resource to the PCPs a behavioral clinician trained in motivational interviewing and brief intervention. The pilot took advantage of the Teen Well Check AOD use questions included in the health system’s EMR to use as “trigger” questions for identifying patients at risk and provided brief training to PCPs on the need for systematic AOD screening and on the process for referring teens to the behavioral clinician. This intervention encouraged a more systematic approach, indirectly addressing the self-reported tendency of PCPs to screen by gender, age, substance type, or other characteristics. Sessions of SBIRT included further screening; brief interventions for substance use, depression, anxiety, and other behavioral risk behaviors for patients with lower-severity AOD problems; and clinician-facilitated referral and follow-up to specialty AOD and mental health treatment as needed for patients with higher-severity AOD or mental health problems. To assess whether the intervention resulted in more screening and intervention, we used the EMR to compare pre- and post-intervention rates of screening and brief intervention and AOD and mental health visits. Rates of behavioral-health treatment utilization increased during the pilot; for adolescents with routine well-child visits, specialty behavioral health treatment initiation increased from 8.7% to 12.0% (p < 0.0001). Although the findings from this small pilot cannot be interpreted as causal, and the project did not have the resources to address many of the provider and patient factors identified in the survey as influencing screening practices, the results suggest ways to increase screening for, and treatment of, adolescent AOD problems in pediatric primary care settings. Further research on interventions designed to address provider-level factors that inhibit screening would be beneficial; specifically, enhanced medical and continuing education training on AOD screening and brief intervention and the development of streamlined, provider-friendly instruments and workflows to address adolescent AOD problems.

Because adolescent AOD problems frequently co-occur with other medical and mental health conditions [15,16], and these adolescents are costly consumers of health services [79], the early identification and prevention of AOD problems should be of great interest to policy-makers and health-care systems. Under health-care reform legislation, many health systems are now required to retain members into young adulthood. Future studies should continue to test models of care that can deliver AOD and behavioral health services efficiently and cost-effectively and in a manner to which providers will be amenable.

This study was conducted in a private-sector integrated health system with a relatively mature EMR containing a behavioral health screener. As such, results may not be generalizable to other health systems. However, systems such as KPNC’s have increasingly become a major organizational model for private and public health care, including many state Medicaid systems. Fundamental changes are occurring that provide an incentive to integrate behavioral health into primary care in the US in both public and private settings. These include health reform and parity legislation and a substantial increase in federal funding flowing to safety net providers such as federally qualified health centers (FQHCs) and community health centers to promote the integration of behavioral health services and training and the implementation of EMR [77]. In the coming decade, over half of patients newly insured under health-care reform will be insured through Medicaid with the Children’s Health Insurance Program (CHIP) rolled into it [80], most of whom will receive services in private health systems and FQHCs. Our findings on barriers and facilitators of AOD screening of adolescents in primary care and the potential effectiveness of a systematic adolescent SBIRT model may help to inform this transformation of primary care practice.

## Competing interests

The authors declare that they have no competing interests.

## Authors’ contributions

The authors declare that each contributed equally to writing this manuscript and reviewed and approved the final draft. AKS conducted statistical analysis of the data. The information in this paper was presented in part at the 8th Annual International Network on Brief Interventions for Alcohol Problems (INEBRIA) Conference, September 21–23, 2011, Boston, MA, USA.

## References

[B1] National Institute on Alcohol Abuse and Alcoholism Five-year strategic plan (FY07-11) alcohol across the lifespan http://pubs.niaaa.nih.gov/publications/StrategicPlan/NIAAASTRATEGICPLAN.htm

[B2] Brindis C Park MJ Ozer EM Irwin CE Jr Adolescents' access to health services and clinical preventive health care: crossing the great divide Pediatr Ann 2002 31 575 581 12271742 10.3928/0090-4481-20020901-10

[B3] Blum R Physicians′ assessment of deficiencies and desire for training in adolescent care J Med Educ 1987 62 401 407 3573019 10.1097/00001888-198705000-00005

[B4] Shrier LA Harris SK Kurland M Knight JR Substance use problems and associated psychiatric symptoms among adolescents in primary care Pediatrics 2003 111 e699 e705 12777588 10.1542/peds.111.6.e699

[B5] Knight JR Harris SK Sherritt L Van Hook S Lawrence N Brooks T Carey P Kossack R Kulig J Prevalence of positive substance abuse screen results among adolescent primary care patients Arch Pediatr Adolesc Med 2007 161 1035 1041 17984404 10.1001/archpedi.161.11.1035

[B6] Ammon L Sterling S Mertens J Weisner C Adolescents in private chemical dependency programs: Who are most at risk for HIV? J Subst Abuse Treat 2005 29 39 45 15979530 10.1016/j.jsat.2005.03.003

[B7] US Substance Abuse and Mental Health Services Administration Report to Congress on the Prevention and Treatment of Co-Occurring Substance Use Disorders and Mental Disorders http://www.samhsa.gov/reports/congress2002/CoOccurringRpt.pdf

[B8] Windle M Spear LP Fuligni AJ Angold A Brown JD Pine D Smith GT Giedd J Dahl RE Transitions into underage and problem drinking: developmental processes and mechanisms between 10 and 15 years of age Pediatrics 2008 121 Suppl 4 S273 S289 18381494 10.1542/peds.2007-2243C PMC2892675

[B9] Brown SA Tapert SF Granholm E Delis DC Neurocognitive functioning of adolescents: effects of protracted alcohol use Alcohol Clin Exp Res 2000 24 164 171 10698367

[B10] Merenstein D Green L Fryer GE Dovey S Shortchanging adolescents: room for improvement in preventive care by physicians Fam Med 2001 33 120 123 11271739

[B11] Levy S Vaughan BL Knight JR Office-based intervention for adolescent substance abuse Pediatr Clin North Am 2002 49 329 343 11993286 10.1016/s0031-3955(01)00007-4

[B12] Yoast RA Fleming M Balch GI Reactions to a concept for physician intervention in adolescent alcohol use J Adolesc Health 2007 41 35 41 17577532 10.1016/j.jadohealth.2007.02.008 PMC2001271

[B13] Brown JD Wissow LS Discussion of sensitive health topics with youth during primary care visits: relationship to youth perceptions of care J Adolesc Health 2009 44 48 54 19101458 10.1016/j.jadohealth.2008.06.018 PMC2630026

[B14] Freeborn DK Polen MR Mullooly JP Adolescent drug misuse treatment and use of medical care services Int J Addict 1995 30 795 822 PMCID: PMC040599/itn 7558471 10.3109/10826089509067008

[B15] Mertens JR Flisher AJ Fleming MF Weisner CM Medical conditions of adolescents in alcohol and drug treatment: comparison with matched controls J Adolesc Health 2007 40 173 179 17259058 10.1016/j.jadohealth.2006.09.021 PMC1876784

[B16] Sterling S Kohn CS Lu Y Weisner C Pathways to chemical dependency treatment for adolescents in an HMO J Psychoactive Drugs 2004 36 439 453 15751482 10.1080/02791072.2004.10524427

[B17] Levy SJ Kokotailo PK Substance use screening, brief intervention, and referral to treatment for pediatricians Pediatrics 2011 128 e1330 e1340 22042818 10.1542/peds.2011-1754

[B18] Kaul P Coupey SM Clinical evaluation of substance abuse Pediatr Rev 2002 23 85 94 11875181 10.1542/pir.23-3-85

[B19] Knight JR The role of the primary care provider in preventing and treating alcohol problems in adolescents Ambul Pediatr 2001 1 150 161 11888392 10.1367/1539-4409(2001)001<0150:trotpc>2.0.co;2

[B20] American Academy of Pediatrics/Bright Futures Recommendations for preventive pediatric health care. Periodicity Schedule. Practice Management Onlinehttp://practice.aap.org/content.aspx?aid=1599 10.1542/peds.2015-2009 26324870

[B21] American Academy of Pediatrics Committee on Substance Abuse Tobacco, alcohol, and other drugs: The role of the pediatrician in prevention and management of substance abuse Pediatrics 1998 101 125 128 11345974

[B22] American Medical Association Guidelines for Adolescent Preventive Services (GAPS) 1997 Chicago: American Medical Association 10.1001/archpedi.1997.02170460096021 9308880

[B23] US Preventive Services Task Force Guide to Clinical Preventive Services, AHRQ Publication No. 08–05122 2008 http://odphp.osophs.dhhs.gov/pubs/guidecps/pcpstoc.htm

[B24] American Academy of Pediatrics Research update: 45 % of fellows routinely screen for alcohol usehttp://aapnews.aappublications.org/cgi/content/short/14/10/1

[B25] Bethell C Klein J Peck C Assessing health system provision of adolescent preventive services: The Young Adult Health Care Survey Med Care 2001 39 478 490 11317096 10.1097/00005650-200105000-00008

[B26] Friedman LS Johnson B Brett AS Evaluation of substance-abusing adolescents by primary care physicians J Adolesc Health Care 1990 11 227 230 2358391 10.1016/0197-0070(90)90353-4

[B27] Emans SJ Bravender TD Knight JR Frazer C Luoni M Berkowitz C Armstrong E Goodman E Adolescent medicine training in pediatric residency programs: Are we doing a good job? Pediatrics 1998 102 588 595 9738181 10.1542/peds.102.3.588

[B28] Spandorfer JM Israel Y Turner BJ Primary care physicians' views on screening and management of alcohol abuse: inconsistencies with national guidelines J Fam Pract 1999 48 899 902 10907628

[B29] Steiner BD Gest KL Do adolescents want to hear preventive counseling messages in outpatient settings? J Fam Pract 1996 43 375 381 8874373

[B30] Friedmann PD McCullough D Chin MH Saitz R Screening and intervention for alcohol problems. A national survey of primary care physicians and psychiatrists J Gen Intern Med 2000 15 84 91 10672110 10.1046/j.1525-1497.2000.03379.x PMC1495340

[B31] Ozer EM Adams SH Gardner LR Mailloux DE Wibbelsman CJ Irwin CE Jr Provider self-efficacy and the screening of adolescents for risky health behaviors J Adolesc Health 2004 35 101 107 15261638 10.1016/j.jadohealth.2003.09.016

[B32] Gottlieb NH Mullen PD McAlister AL Patients′ substance abuse and the primary care physician: patterns of practice Addict Behav 1987 12 23 32 3565109 10.1016/0306-4603(87)90005-0

[B33] Wilson CR Sherritt L Gates E Knight JR Are clinical impressions of adolescent substance use accurate? Pediatrics 2004 114 e536 e540 15520086 10.1542/peds.2004-0098

[B34] Stevens J Kelleher KJ Gardner W Chisolm D McGeehan J Pajer K Buchanan L Trial of computerized screening for adolescent behavioral concerns Pediatrics 2008 121 1099 1105 18519478 10.1542/peds.2007-1878

[B35] Hassan A Harris SK Sherritt L Van Hook S Brooks T Carey P Kossack R Kulig J Knight JR Primary care follow-up plans for adolescents with substance use problems Pediatrics 2009 124 144 150 19564294 10.1542/peds.2008-2979 PMC4103426

[B36] Monti PM Colby SM Barnett NP Spirito A Rohsenow DJ Myers M Woolard R Lewander W Brief intervention for harm reduction with alcohol-positive older adolescents in a hospital emergency department J Consult Clin Psychol 1999 67 989 994 10596521 10.1037//0022-006x.67.6.989

[B37] Gates S McCambridge J Smith LA Foxcroft DR Interventions for prevention of drug use by young people delivered in non-school settings Cochrane Database Syst Rev Issue 1. Art. No.: CD000353 2004 10.1002/14651858.CD000353.pub2 PMC13222737 16437511

[B38] Spirito A Monti PM Barnett NP Colby SM Sindelar H Rohsenow DJ Lewander W Myers M A randomized clinical trial of a brief motivational intervention for alcohol-positive adolescents treated in an emergency department J Pediatr 2004 145 396 402 15343198 10.1016/j.jpeds.2004.04.057

[B39] Winters KC Leitten W Wagner E O’Leary Tevyaw T Use of brief interventions for drug abusing teenagers within a middle and high school setting J Sch Health 2007 77 196 206 17425522 10.1111/j.1746-1561.2007.00191.x

[B40] Knight JR Sherritt L Van Hook S Gates EC Levy S Chang G Motivational interviewing for adolescent substance use: a pilot study J Adolesc Health 2005 37 167 169 16026730 10.1016/j.jadohealth.2004.08.020

[B41] Leontieva L Horn K Haque A Helmkamp J Ehrlich P Williams J Carroll KM Ball SA Nich C Martino S Frankforter TL Farentinos C Kunkel LE Mikulich-Gilbertson SK Morgenstern J Obert JL Polcin D Snead N Woody GE Borsari B Carey KB Freyer J Tonigan JS Keller S Rumpf HJ John U Hapke U Readiness to change problematic drinking assessed in the emergency department as a predictor of change J Crit Care 2005 20 251 256 16253794 10.1016/j.jcrc.2005.05.009

[B42] Lawendowski LA A motivational intervention for adolescent smokers Prev Med 1998 27 A39 A46 9808816 10.1006/pmed.1998.0424

[B43] Martin G Copeland J Swift W The Adolescent Cannabis Check-Up: feasibility of a brief intervention for young cannabis users J Subst Abuse Treat 2005 29 207 213 16183469 10.1016/j.jsat.2005.06.005

[B44] Marlatt GA Baer JS Kivlahan DR Dimeff LA Larimer ME Quigley LA Somers JM Williams E Screening and brief intervention for high-risk college student drinkers: results from a two-year follow-up assessment J Consult Clin Psych 1998 66 604 615 10.1037//0022-006x.66.4.604 9735576

[B45] Bernstein J Heeren T Edward E Dorfman D Bliss C Winter M Bernstein E A brief motivational interview in a pediatric emergency department, plus 10-day telephone follow-up, increases attempts to quit drinking among youth and young adults who screen positive for problematic drinking Acad Emerg Med 2010 17 890 902 20670329 10.1111/j.1553-2712.2010.00818.x PMC2913305

[B46] Walton MA Chermack ST Shope JT Bingham CR Zimmerman MA Blow FC Cunningham RM Effects of a brief intervention for reducing violence and alcohol misuse among adolescents: a randomized controlled trial JAMA 2010 304 527 535 20682932 10.1001/jama.2010.1066 PMC3560393

[B47] Baer JS Kivlahan DR Blume AW McKnight P Marlatt GA Brief intervention for heavy-drinking college students: 4-year follow-up and natural history Am J Public Health 2001 91 1310 1316 11499124 10.2105/ajph.91.8.1310 PMC1446766

[B48] Borsari B Carey KB Effects of a brief motivational intervention with college student drinkers J Consult Clin Psychol 2000 68 728 733 10965648

[B49] Carey KB Correia CJ Drinking motives predict alcohol-related problems in college students J Stud Alcohol 1997 58 100 105 8979218 10.15288/jsa.1997.58.100

[B50] Monti PM Barnett NP Colby SM Gwaltney CJ Spirito A Rohsenow DJ Woolard R Motivational interviewing versus feedback only in emergency care for young adult problem drinking Addiction 2007 102 1234 1243 17565560 10.1111/j.1360-0443.2007.01878.x

[B51] Larimer ME Turner AP Anderson BK Fader JS Kilmer JR Palmer RS Cronce JM Evaluating a brief alcohol intervention with fraternities J Stud Alcohol 2001 62 370 380 11414347 10.15288/jsa.2001.62.370

[B52] Murphy JG Duchnick JJ Vuchinich RE Davison JW Karg RS Olson AM Smith AF Coffey TT Relative efficacy of a brief motivational intervention for college student drinkers Psychol Addict Behav 2001 15 373 379 11767271 10.1037//0893-164x.15.4.373

[B53] Tait RJ Hulse GK Robertson SI Sprivulis PC Emergency department-based intervention with adolescent substance users: 12-month outcomes Drug Alcohol Depend 2005 79 359 363 16102378 10.1016/j.drugalcdep.2005.03.015

[B54] Ozer E Lustig JL Adams S Gee AGS Wibblesman C Bonar RW Fuster D Irwin J Charles J Integrating training into practice: Increasing the delivery of adolescent clinical preventive services J Adolesc Health 2003 32 130 131

[B55] De Micheli D Fisberg M Formigoni ML Study on the effectiveness of brief intervention for alcohol and other drug use directed to adolescents in a primary health care unit Rev Assoc Med Bras 2004 50 305 313 15499485 10.1590/s0104-42302004000300040

[B56] D’Amico EJ Miles JN Stern SA Meredith LS Brief motivational interviewing for teens at risk of substance use consequences: a randomized pilot study in a primary care clinic J Subst Abuse Treat 2008 35 53 61 18037603 10.1016/j.jsat.2007.08.008

[B57] Aira M Kauhanen J Larivaara P Rautio P Factors influencing inquiry about patients' alcohol consumption by primary health care physicians: qualitative semi-structured interview study Fam Pract 2003 20 270 275 12738695 10.1093/fampra/cmg307

[B58] Rowland N Maynard A Beveridge A Kennedy P Wintersgill W Doctors have no time for alcohol screening Br Med J 1987 295 95 96 3113652 10.1136/bmj.295.6590.95-a PMC1246969

[B59] Yarnall KS Pollak KI Ostbye T Krause KM Michener JL Primary care: is there enough time for prevention? Am J Public Health 2003 93 635 641 12660210 10.2105/ajph.93.4.635 PMC1447803

[B60] Horwitz SM Kelleher KJ Stein RE Storfer-Isser A Youngstrom EA Park ER Heneghan AM Jensen PS O'Connor KG Hoagwood KE Barriers to the identification and management of psychosocial issues in children and maternal depression Pediatrics 2007 119 e208 e218 17200245 10.1542/peds.2005-1997

[B61] Van Hook S Harris SK Brooks T Carey P Kossack R Kulig J Knight JR The “Six T′s”: barriers to screening teens for substance abuse in primary care J Adolesc Health 2007 40 456 461 17448404 10.1016/j.jadohealth.2006.12.007

[B62] Meadows T Valleley R Haack MK Thorson R Evans J Physician “costs” in providing behavioral health in primary care Clin Pediatr (Phila) 2011 50 447 455 21196418 10.1177/0009922810390676

[B63] Stein RE Horwitz SM Storfer-Isser A Heneghan A Olson L Hoagwood KE Do pediatricians think they are responsible for identification and management of child mental health problems? Results of the AAP periodic survey Ambul Pediatr 2008 8 11 17 18191776 10.1016/j.ambp.2007.10.006

[B64] Friedmann PD McCullough D Saitz R Screening and intervention for illicit drug abuse: a national survey of primary care physicians and psychiatrists Arch Intern Med 2001 161 248 251 11176739 10.1001/archinte.161.2.248

[B65] Ewing GB Selassie AW Lopez CH McCutcheon EP Self-report of delivery of clinical preventive services by U.S. physicians. Comparing specialty, gender, age, setting of practice, and area of practice Am J Prev Med 1999 17 62 72 10429755 10.1016/s0749-3797(99)00032-x

[B66] Klein JD Wilson KM Delivering quality care: adolescents' discussion of health risks with their providers J Adolesc Health 2002 30 190 195 11869926 10.1016/s1054-139x(01)00342-1

[B67] National Center on Addiction and Substance Abuse at Columbia University Missed opportunity: National Survey of Primary Care Physicians and Patients on Substance Abuse [http://www.casacolumbia.org/articlefiles/380-Missed%20Opportunity%20Physicians%20and%20Patients.pdf]

[B68] Gardner W Pajer KA Kelleher KJ Scholle SH Wasserman RC Child sex differences in primary care clinicians' mental health care of children and adolescents Arch Pediatr Adolesc Med 2002 156 454 459 11980550 10.1001/archpedi.156.5.454

[B69] Gardner W Kelleher KJ Pajer KA Campo JV Primary care clinicians’ use of standardized tools to assess child psychosocial problems Ambul Pediatr 2003 3 191 195 12882596 10.1367/1539-4409(2003)003<0191:pccuos>2.0.co;2

[B70] Weisner C Matzger H Missed opportunities in screening for alcohol problems in medical and mental health services Alcohol Clin Exp Res 2003 27 1132 1141 12878919 10.1097/01.ALC.0000075546.38349.69

[B71] Volk RJ Steinbauer JR Cantor SB Patient factors influencing variation in the use of preventive interventions for alcohol abuse by primary care physicians J Stud Alcohol 1996 57 203 209 8683970 10.15288/jsa.1996.57.203

[B72] McCrady BS Richter SS Morgan TJ Slade J Pfeifer C Involving health care workers in screening for alcohol problems J Addict Dis 1996 15 45 58 8842849 10.1300/J069v15n03_03

[B73] Franzgrote M Ellen JM Millstein SG Irwin CE Jr Screening for adolescent smoking among primary care physicians in California Am J Public Health 1997 87 1341 1345 9279272 10.2105/ajph.87.8.1341 PMC1381097

[B74] Ellen JM Franzgrote M Irwin CE Jr Millstein SG Primary care physicians’ screening of adolescent patients: a survey of California physicians J Adolesc Health 1998 22 433 438 9627812 10.1016/s1054-139x(97)00276-0

[B75] Friedmann PD Saitz R Gogineni A Zhang JX Stein MD Validation of the screening strategy in the NIAAA “Physicians’ Guide to Helping Patients with Alcohol Problems J Stud Alcohol 2001 62 234 238 11332444 10.15288/jsa.2001.62.234

[B76] Institute of Medicine Improving the Quality of Health Care for Mental and Substance-Use Conditions: Quality Chasm Series 2006 Washington, DC: National Academies Press 20669433

[B77] Mental Health America Parity and health care reform: important changes for behavioral health http://www.nmha.org/action/webinars/2010-07/Parity_and_HCR_July-8-2010.pdf

[B78] Mignone J Washington R Ramesh BM Blanchard JF Rajaretnam T Moses S Discrepancies between the self-reporting of STI preventive care and the actual care provided by male doctors to male patients in Karnataka, India Sex Transm Infect 2010 86 391 392 20682562 10.1136/sti.2009.038851

[B79] Parthasarathy S Weisner C Health care services use by adolescents with intakes into an outpatient alcohol and drug treatment program Am J Addict 2006 15 Suppl 113 121 17182426 10.1080/10550490601006097

[B80] Sisko AM Truffer CJ Keehan SP Poisal JA Clemens MK Madison AJ National health spending projections: the estimated impact of reform through 2019 Health Aff (Millwood) 2010 29 1933 1941 20829295 10.1377/hlthaff.2010.0788

